# Targeting of the Essential *acpP*, *ftsZ*, and *rne* Genes in Carbapenem-Resistant *Acinetobacter baumannii* by Antisense PNA Precision Antibacterials

**DOI:** 10.3390/biomedicines9040429

**Published:** 2021-04-15

**Authors:** Alireza Japoni Nejad, Nader Shahrokhi, Peter E. Nielsen

**Affiliations:** 1 Center for Peptide-Based Antibiotics, Department of Cellular and Molecular Medicine, Faculty of Health and Medical Sciences, The Panum Institute, University of Copenhagen Blegdamsvej 3, DK-2200 Copenhagen, Denmark; alireza@sund.ku.dk; 2 Department of Molecular Biology, Pasteur Institute of Iran, Pasteur Ave, Tehran 13164, Iran; Shahrokhi@pasteur.ac.ir

**Keywords:** precision antibiotics, antisense, *A. baumannii*, carbapenem resistance, peptide nucleic acid (PNA)

## Abstract

Infections by carbapenem-resistant *A. baumannii* (CRAB), a widespread nosocomial pathogen, are becoming increasingly difficult to prevent and treat. Therefore, there is an urgent need for discovery of novel antibiotics against CRAB. Programmable, precision antisense antibiotics, e.g., based on the nucleic acid mimic PNA (peptide nucleic acid) have shown promise in this respect in the form of PNA-BPP (bacteria penetrating peptide) conjugates targeting essential bacterial genes. In the present study, we designed and synthesized a series of PNA-BPPs targeting the translation initiation region of the *ftsZ*, *acpP*, or *rne* gene of CRAB strains. The antimicrobial activity of the compounds and effects on gene expression level was compared to that of analogous mismatch PNA controls. Three antisense conjugates (KFF)_3_K-eg1-(*acpP*)PNA (5639), (KFF)_3_K-eg1-(*ftsZ*)PNA (5612), and (KFF)_3_-K-eg1-(*rne*)PNA (5656) exhibited complete growth inhibition against several CRAB strains at 1–2, 2–8, and 2 µM, respectively, and the compounds were bactericidal at 1–2× MIC. The bactericidal effect was correlated to reduction of target gene mRNA level using RT-qPCR, and the compounds showed no bacterial membrane disruption activity at 1–2× MIC. PNA5612 was tested against a series of 12 CRAB isolates and all were sensitive at 2–8 µM. In addition, the conjugates exhibited no cellular toxicity in the HepG2 cell line (up to 20 μM) and did not shown significant antibacterial activity against other Gram negatives (*E. coli*, *P. aeruginosa*). These results provide a starting point for discovery of antisense precision designer antibiotics for specific treatment of CRAB infections.

## 1. Introduction

*Acinetobacter baumannii* is a major cause of both community-associated and nosocomial infections that are difficult to control and treat worldwide, and antibiotic resistance is becoming a major problem in hospitals where outbreaks are reported frequently, especially in intensive care units [1,2]. *A. baumannii* has a remarkable ability to rapidly develop antibiotic resistance, which has led to multidrug resistant strains within a few decades [3]. Presently, a substantial proportion of these isolates are also carbapenem resistant, and the mortality rates for the most common carbapenem resistant *A. baumannii* (CRAB) infections, primarily hospital-acquired pneumonia and bloodstream infections, can approach 60% [4]. Currently used antimicrobials for CRAB (polymyxins and tigecycline) are far from perfect therapeutic options due to toxicity issues and increasing resistance rates also against these antibiotics [3,4]. Thus, discovery of new classes of antibiotics with novel molecular targets and mechanism of action for treating and managing CRAB infections is urgently needed.

Exploitation of antisense antimicrobials in the form of short oligonucleotide analogous and mimics that inhibit that expression of essential genes at the RNA level is a promising approach [5,6], which was introduced two decades ago using 9-12-mer PNA (peptide nucleic acid) oligomers targeting the essential *acpP* gene in *E. coli* [7]. Subsequently, PNA and PMO (phosphorodiamidate morpholino oligomer) antibacterial agents have been studied extensively for targeting a multitude of genes in many bacterial species [8,9]. In theory, this approach allows targeting of any expressed gene, thereby making it druggable, and circumventing known resistance mechanisms. In addition, the intended target bacteria can be programmed specifically via the chosen gene sequence with high precision.

PNA is a charge neutral, hydrophilic synthetic polymer with a pseudo-peptide backbone exhibiting high sequence specific affinity for complementary RNA (and DNA) molecules. In addition, PNA oligomers have very high biological stability being practically inert to both nucleases and peptidases [10], but cellular (including bacterial) uptake is a general challenge in the application of PNAs (as well as PMOs) as antisense agents. However, conjugation to bacteria penetrating peptides (BPPs) was discovered as a successful way to ensure increased PNA uptake and thereby efficacy [7]. In the last two decades, many mRNA encoding essential genes in clinically pathogenic bacteria have been validated as possible targets for antisense antibacterial agents. The most effective antisense agents were designed complementary to the mRNA around or proximally upstream of the translation start codon [11]. Upon binding to the mRNA, PMO and PNA act through steric hindrance of mRNA translation or processing, contrary to some other antisense oligonucleotides that induce mRNA degradation via activation of RNase H.

In this study, we have investigated and characterized antisense specific antimicrobial activity of the series of peptide–PNA conjugates targeting the essential *acpP*, *ftsZ*, and *rne* genes in carbapenem resistant clinical *Acinetobacter baumannii* isolates with the ultimate aim of discovering new precision antibiotics.

## 2. Materials and Methods

### 2.1. Bacterial Strains

Strains used in this study are listed in Table S1-A. Clinical isolates of carbapenem-resistant *A. baumannii* (CRAB) were previously collected from Valiasr hospital (Arak-Iran) [12]. Standard biochemical tests were performed to identify *A. baumannii* phenotypically. In addition, *bla_OXA-51_* PCR was performed. Species-level identification of isolates was reconfirmed by the MALDI Biotyper system (Statens Seruminstitute (SSI), Copenhagen, Denmark). One CRAB strain (SSI1104) was kindly provided by Dr. Anette M. Hammerum (SSI carbapenemase collection, Copenhagen-Denmark). *A. baumannii* ATCC 19606 was used as a reference strain. Clinical isolates were previously tested for the presence of the carbapenemase encoding genes such as *bla_OXA-51_*, *bla_OXA-23_*, *bla_OXA-24_*, *bla_OXA-58_*, *bla_IMP_*, *bla_VIM_*, *bla_SPM_*, *bla_GES_*, *bla_SIM_*, and *bla_GIM_* according to the Ellington study (Table S1B) [13].

### 2.2. Antibiotic MIC

All minimum inhibitory concentration (MIC) determinations for antibiotics were performed by the standard microdilution method in 96-well microplate (Thermo-Scientific, Roskilde, Denmark) or by gradient test strips (Liofilchem, Italy) according to the Clinical and Laboratory Standards Institute guidelines [14]. *Escherichia coli* ATCC 25922 was used as control.

### 2.3. Computational Screening of Sequence Regions in the mRNA of Target Genes

*Acinetobacter baumannii* strains that were examined for designing antisense in this study are shown in Table S2. Sequence alignment of target genes (obtained from GenBank database updated January 2019) were carried out using the Basic Local Alignment Search Tool (BLAST) on the National Center for Biotechnology Information (NCBI) website [15].

### 2.4. BPP-PNA Synthesis

Peptide–PNA conjugates (Tables S3–S5) were synthesized by continuous solid phase synthesis using Boc-chemistry as previously described [7]. The conjugates were purified by reversed phase high-performance liquid chromatography (HPLC) on an RP18 column using a 0–40% acetonitrile gradient in 0.1% trifluoroacetic acid. Characterization in terms of purity and identity was done by HPLC and matrix-assisted laser desorption/ionization-time of flight (MALDI-TOF) mass spectrometric analyses. All conjugates exhibited masses within experimental error corresponding to the calculated mass and were >95% pure by HPLC analysis (Figure S7).

### 2.5. BPP-PNA MIC

MIC values were determined by broth microdilution according to standard protocols with a few modifications. An overnight bacterial cell culture was diluted to approximately 5 × 10^5^ CFU/mL in non-cation-adjusted Muller Hinton Broth (MHB). Then, 180 µL bacterial solutions were dispensed into a low-bind 96-well plate (Thermo-Scientific, Roskilde, Denmark) along with 20 µL of BPP-PNA compound (BPP-PNA was serially diluted 2-fold from 16 to 0.5 µM in a 96-well plate). The plate was incubated in a Tecan Genios plate reader (GMI, Minnesota, Ramsey, USA) at 37 °C for 18 h with linear shaking, OD was measured every 20 min at 595 nm. The MIC was determined as the lowest concentration, that inhibited visible growth (OD < 0.1) in the wells [16]. The minimum bactericidal concentration (MBC) values were determined by plating 10 µL samples from the wells with no visible turbidity onto Mueller–Hinton agar plates. The MBC is determined the lowest concentration of BPP-PNA that reduces the viability of the initial bacterial inoculum by ≥99.9% (3-log reduction in CFU/mL). A minimum of two independent experiments (biological replicates) of the assay were conducted, and two technical replicates were used in each experiment for each bacterium, compound, and concentration.

### 2.6. Time-Kill Assay

*A. baumannii* strains in logarithmic growth phase were diluted to ~10^6^ CFU/mL and incubated with different concentration (corresponding to the MIC-value) of the BPP-PNA at 37 °C for 6 h.

Bacterial cultures were harvested at 0, 90 min, 3 and 6 h. The content of each well was added to 0.9 mL of MHB and centrifuged at 9300× *g* for 5 min. Supernatants were removed and the cell pellets were suspended in fresh pre-warmed MHB (100 μL). Tenfold serial dilution were prepared, and 10 μL aliquots were plated on Mueller–Hinton agar and incubated overnight at 37 °C to determine the CFU/mL [17].

### 2.7. HepG2 Cell Toxicity

The cell toxicity of selected BPP-PNAs was determined using HepG2 hepatocytes grown in DMEM (low glucose) medium supplemented with 100 U/mL penicillin, 100 µg/mL streptomycin, 1% glutamax, and 10% fetal bovine serum (FBS) (Thermofisher Scientific, Roskilde, Denmark), at 37 °C with 5% CO_2_. Then, 7000–10,000 cells were seeded in each well of a 96-well plate. After 48 h, the BPP-PNAs were added (six replicates for each concentration), followed by overnight incubation. Total amount of adenosine triphosphate (ATP) was used as a measure of cell viability, according to the CellTiter-Glo luminescent cell viability assay (Promega, Madison, WI, USA) kit. BPP-PNAs concentrations ranged from 2.5 to 20 µM. Potassium dichromate (100 µM) was used as a positive control.

### 2.8. Envelope Disruption

A culture of *A. baumannii* ATCC 19606 was grown in MHB (195 rpm at 37 °C), until OD595 = 0.4. Then, bacterial cells were pelleted by centrifugation, washed with 0.9% NaCl, and resuspended in MHB at an OD595 = 0.1. Sytox green dye (Invitrogen) was added to the bacterial culture at a concentration of 2 µM followed by incubation for 5 min at 37 °C, followed by addition of the desired amount of BPP–PNA in a 96-well plate format. Fluorescence intensity (λex = 480 nm, λem = 520 nm) was measured every 5 min for 90 min at 37 °C with continuous shaking. Colistin (Sigma Aldrich, Merck, Darmstadt, Germany) at 1× and 2× MIC concentrations provided the positive control [18].

### 2.9. Real-Time RT-PCR

Real time RT-PCR was used to determine the relative expression level of the target gene upon treatment with match BPP-PNA compared to mismatch and untreated controls. The primers for q-PCR were designed to match conserved gene sequences using the AlleleID7 software (Premier Biosoft, San Francisco, CA, USA) (Table S6). Additionally, for more accurate expression of the genes at treatment and untreated conditions, the efficacy of target gene and internal controls primers was calculated by using three consecutive dilutions of the sample cDNA and drawing a standard curve through the slope of the line. NormFinder software was used to select the most stable reference gene among *polA*, *zipA*, and *16S rRNA.* Statistical analysis was carried out on the gene expression levels normalized to the chosen reference gene (16S rRNA) through −∆Ct analysis. The relative expression of each gene was determined by the 2^−ΔΔCT^ method [19]. We used the GenEx standard (NormFinder and GeNorm) software (bioMCC, Germany) for analyzing the data.

An overnight culture of bacteria was diluted 1:100 in MHB medium and was grown with shaking (160 rpm) at 37 °C to reach the OD595 = 0.2. Then, the bacterial culture was treated with different concentrations of BPP-PNA for 1 h and the bacterial cells were harvested by centrifugation and the cell pellet used for RNA extraction [19].

Total RNA was isolated using the GeneJET RNA purification kit (ThermoFisher Scientific, Roskilde, Denmark) following the manufacturer’s protocol. RNA samples were treated with DNase for 30 min at 37 °C (Turbo DNA-free kit Ambion/Applied Biosystems, Carlsbad, CA, USA) and then incubated at 72 °C for 5 min to inactivate the DNase. The genomic DNA (gDNA) contamination was checked by polymerase chain reaction (PCR). RNA concentrations were determined by Nanodrop spectrophotometry at 260 nm and purity was checked by determining the absorption ratios at 260/280 nm. Total RNA (0.5 μg) was reverse-transcribed to yield cDNA as template for real-time quantitative PCR using the Quanti Tect reverse transcription kit (Qiagen, Copenhagen, Denmark). The cDNA was diluted 1:50 and used as a template in the reaction. The real-time polymerase chain reaction was carried out with 5 µL of cDNA in a 20 μL reaction consisting of 4 µL Light Cycler Fast Start DNA Master SYBER I Green (Roche), 0.5 µL of each primer (final concentration = 0.25 µM), and 10 µL of water using Biorad CFX96 TouchTM real-time PCR detection system (Bio-Rad Laboratories Inc., Hercules, CA, USA) under these conditions: pre-incubation at 95 °C for 8 min, followed by 40 cycles with denaturing at 95 °C for 10 s, annealing at 50 °C for 15 s, and elongation at 72 °C for 15 s and then 8 min final extension.

### 2.10. Statistics

All statistical analyses were carried out in SPSS 17.0 (SPSS, Chicago, IL, USA). Data are expressed as mean ± standard deviation (SD) of the biological replicates and analyzed using Student’s *t*-test and ANOVA. *p* values of ≤0.05 were considered significant.

## 3. Results

### 3.1. PNA Design for the mRNA of Target Genes

#### 3.1.1. AcpP

**Figure 1:**
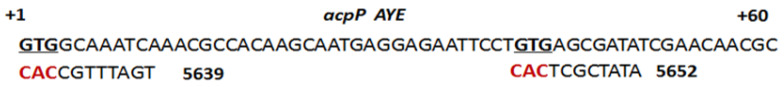
Sequence from the acpP mRNA and positions of the PNA targets.

The *acpP* gene is the original and to date most frequently used antibacterial antisense target [7,16,17,20,21,22]. When searching the *acpP* gene in the NCBI (GenBank) and Genolist databases, two lengths for this gene in different strains of *A. baumannii* were discovered (Figures S2 and S4). The *acpP* coding gene in *A. baumannii* ATCC19606, AYE, and SDF strains was 276 bp in length, while the length was 237 bp for both ACICU and ATCC17978 strains. The difference was related to 39 bases in the first part of the gene utilizing either of two translation initiation sites (GTG at +1 or +40). Therefore, we designed PNAs targeting both of these sites (Figure 1, Table 1).

#### 3.1.2. FtsZ

The *ftsZ* gene has frequently been used as an antibacterial antisense target [17,21,22] and it is well conserved across *A. baumannii* species. BLAST results confirmed that the 5′ terminal region of the *ftsZ* gene including the ribosomal binding site and the start ATG codon is conserved among *A. baumannii* species (Figure S1-A). *A. baumannii* strains and target genes that were examined for designing antisense in this study are shown in Table S2.

#### 3.1.3. RNase E

RNase E is an essential bacterial endoribonuclease involved in the turnover of messenger RNA and the maturation of structured RNA precursors (rRNA and tRNA processing) in *E. coli* [23,24]. The major initiator of bacterial mRNA decay is considered to be a multi-protein complex termed the RNA degradosome. This complex is best characterized in the Gram-negative model organism, *Escherichia coli*, and consists of at least four subunits: RNA helicase B (RhlB), enolase, polynucleotide phosphorylase (PNPase), and RNase E. RNase E is the central component of the *E. coli* degradosome, establishing a scaffold for the assembly of other degradosome subunits and performing the initial endoribonucleolytic event during substrate mRNA decay. Thus, RNase E may represent a promising novel multi-function antimicrobial target. However, this enzyme has so far not been exploited for antisense antibiotic discovery.

In the genome of *A. baumannii* AYE strain, the *rne* gene encodes the 1110-amino acid protein (ABAYE3375). The BlastP analysis revealed an amino acid sequence identity of 60.5% with the *E. coli* RNase E. Thus, there is significant homology between RNase E in *E. coli* and RNase E in *A. baumannii*, and in addition the *rne* gene is well conserved across *A. baumannii* species. The BLAST results confirmed that the 5′ terminal region of the *rne* gene including the ribosomal binding site and the ATG start codon is conserved among *A. baumannii* species (Figure S1-B). In this study, four 11-mer targets overlapping the translation start were chosen.

### 3.2. MIC and Time Kill Kinetics

**Figure 2:**
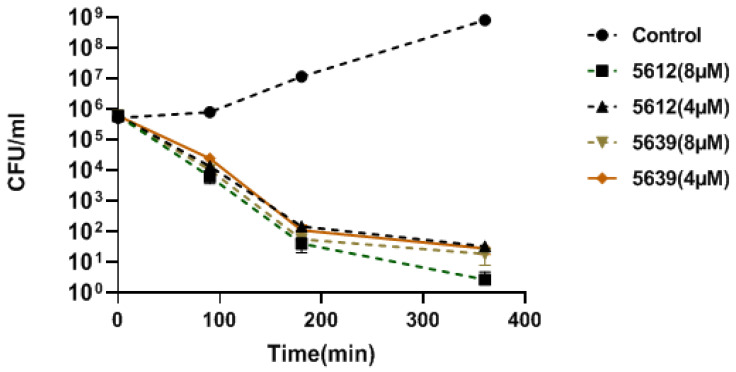
Killing kinetics of anti-*acpP* peptide-PNA (5639) and anti-*ftsZ* peptide-PNA (5612) conjugates against *A. baumannii* ATCC 19606. Strain was grown in the absence of peptide–PNA conjugate (control) or in the presence of MIC and 2× MIC of anti-*acpP* peptide-PNA (5639) and anti-*ftsZ* peptide-PNA (5612). Each point value represents the mean of two independent experiments with at least two technical replicates for each experiment. MHB without cation (Sigma) was used.

We evaluated the antibacterial activity of the compounds by determining the minimum inhibitory concentration (MIC) against several *A. baumannii* strains. The target positions, PNA sequences, CPP, and MIC values are presented below. In case of *acpP*, according to the variation in gene size (*vide supra*), we designed the antisense oligomers to cover both possible start codon regions (Table 1). The MIC value for PNA 5652 targeting the “downstream” GTG was 4 µM in all tested strains, but importantly, when an analogous mismatch PNA control (5653) was tested, no difference in activity was found when compared with the match PNA (5652) (Table 1). Thus, the antibacterial activity could not be assigned to an antisense mechanism of action. However, significant difference between the MIC values of PNA 5639 (targeting the upstream GTG start codon) was found compared with the analogous mismatch control PNA (5640) (Table 1, Figure 1, Figure S5-A). In addition, bactericidal activity of PNA 5639 was characterized against *A. baumannii* ATCC 19606 strain. The ATCC 19606 strain was exposed to 4 or 8 μM of PNA 5639 (and control) for 6 h. As shown in Figure 2, this compound showed fast bactericidal activity resulting in a 4 log reduction after 3 h treatment. The potency of this compound was 1–4-fold decreased when tested in cation adjusted MHB (because divalent cations can stabilize bacterial membranes). Finally, changing the BPP form the natural L-form to the D-form (PNAs 5823 and 5824) reduced both activity and antisense (match/mismatch) specificity.

**Figure 3:**
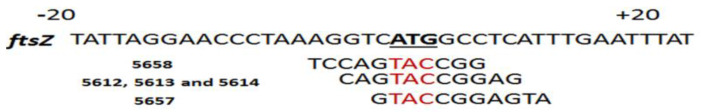
Sequence from the ftsZ mRNA and positions of the PNA targets.

PNA 5612 (Figure 3) was the most active of the anti *ftsZ* PNAs. The MIC values for this compound ranged from 4 to 8 µM and was 2–4-fold lower than for the corresponding mismatch control PNA (5615) (Table 2), corroborating an antisense mechanism of action. The bactericidal activity of PNA 5612 was characterized against the *A. baumannii* ATCC 19606, and as shown in Figure 2, the compound exhibited fast bactericidal activity yielding more than three order of magnitude CFU reduction after 3 h at 4 μM. Furthermore, PNA5612 showed good activity against 12 multidrug resistant clinical isolates with MIC values ranging from 2–8 μM and exhibiting a 2–8-fold match/mismatch ratio (Table 3, Figure S5-B). Finally, exchanging the (KFF)_3_K BPP for the (R-Ahx-R)_4_ BPP, which has been used successfully e.g., in *E. coli* and *Pseudomonas aeruginosa* [17] yielded a significantly less potent compound (PNA5613, MIC > 16M) (Table 2).

**Figure 4:**
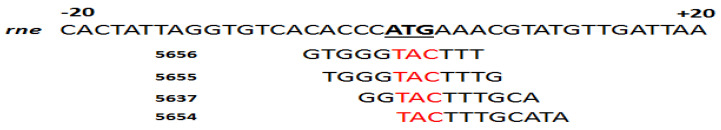
Sequence from the rne mRNA and positions of the PNA targets.

**Figure 5:**
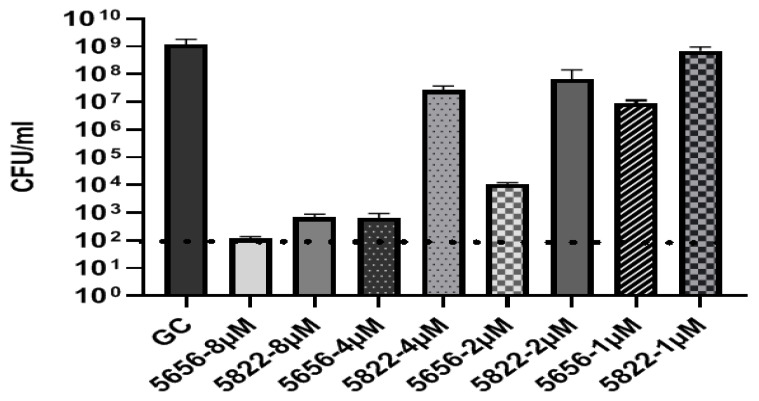
Minimum bactericidal concentration (MBC) assays for the BPP-anti-*rne* PNA (5656) and mismatch control (5822) in antibiotic resistant clinical isolate AC46 after 24 h incubation and concentration range from 1–8 μM. Dashed line represents the limit of detection.

Finally, for the *rne* gene, four different (KFF)_3_K-PNAs were design targeting the start codon in different registers (Figure 4, Table 4). MIC determination showed that PNA5656 (−5 to +6 register) was the most active, yielding a MIC of 2 μM and a four-fold mismatch discrimination. Concentration dependent bactericidal activity was determined on the highly antibiotic resistant clinical isolate AC46 in a concentration range from 1–8 μM (Figure 5) and showed very significant CFU reduction by the match PNA compared to the corresponding mismatch control PNA (5822), thus supporting that *rne* gene expression is essential for *A. baumannii* survival.

### 3.3. Species-Selective Activity of BPP-PNAs

In principle, a specific antisense PNA-peptide conjugate should only show activity against species that harbor the sequence target for the PNA in a sensitive essential gene position (e.g., overlapping the ribosome binding site and/or the translation initiation codon), and in which the conjugate is taken up. Thus, the *A. baumannii* active PNAs 5612, 5639, and 5652 were also tested for activity against *E. coli* and *P. aeruginosa* as a non-target species where no obvious gene target match for the PNAs exists. The results (Table 5) also clearly show that neither of these PNAs are active against *E. coli* and *P. aeruginosa*, thereby illustrating species specific/selective activity of these precision antisense antibiotics. In addition, a fourth analogous PNA (3965) without obvious target in any of the three species likewise did not show activity against these either.

### 3.4. Cytotoxicity

**Figure 6:**
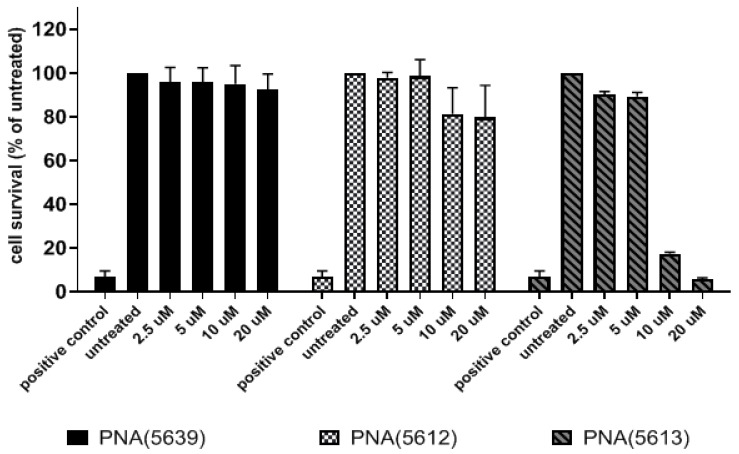
Cell viability assay of anti-*acpP* (KFF)_3_K-PNA 5639, anti-*ftsZ* (KFF)_3_K-PNA 5612, and anti-*ftsZ*(R-Ahx-R)_4_-Ahx-PNA 5613 in HepG2 cell culture. Cell viability was quantified by ATP level. The data are presented as the mean of normalized percentage of two individual experiments (each comprising six replicates for each concentration) relative to untreated cells (error bars represent SEM). Potassium dichromate (100 µM) was used as a positive control.

It is well established that cationic peptides in general can exhibit mammalian cellular toxicity. Thus, a toxicity characterization was performed in a standard in vitro HepG2 cell culture assay based on mitochondrial activity. These results showed significant difference in eukaryotic cell toxicity between PNA 5612 and 5639 compared to PNA 5613 (Figure 6). After 24 h of exposure to 20 µM of BPP-PNAs, the highest concentrations of BPP-PNAs tested resulted in 8–20% reduction in cell viability for BPP-PNAs 5639 and 5612, while exposure to the MIC50 concentration (2–4 µM) resulted in no reduction in viability for both PNAs 5639 and 5612. Notably, the H-(R-Ahx-R)_4_-Ahx-PNA 5613 showed very significant toxicity at 10 µM (EC50 was between 5 and 10 µM).

### 3.5. Envelope Disruption

**Figure 7:**
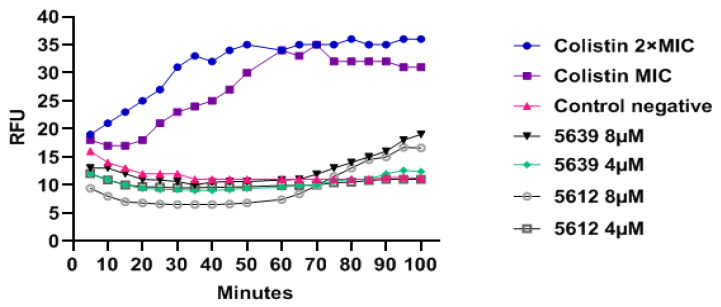
Membrane disruption Sytox assay in A. baumannii ATCC 19606. Membrane disruption activity of anti-*acpP* (KFF)_3_K-PNA 5639 and anti-*ftsZ* (KFF)_3_K-PNA 5612 was measured by fluorescence intensity of internalized Sytox. Colistin was used as positive control.

Structurally BPPs are related to antimicrobial peptides containing both cationic and hydrophobic amino acids (exemplified by lysine and arginine, and phenylalanine and amino hexanoic acid in the peptides used here) and some antimicrobial peptides may even be employed as BPPs [20,25]. Therefore, non-antisense-related activity as seen for some of the BPP-PNAs in this study could be due to membrane/envelope disruption. This may be tested using the DNA binding fluorescent dye Sytox, which is not taken up by live, intact bacteria. However, cell envelope disruption leads to cellular uptake of the Sytox, resulting in an enhanced fluorescence due to DNA binding of the dye which can be observed in both a concentration- and time-dependent manner. Colistin, a known membrane-disrupting agent, was used as a positive control. However, the Sytox uptake assay gave no indication of membrane disruption activity of the anti-*acpP* (KFF)_3_-K-PNA 5639 and anti-*ftsZ* (KFF)_3_-K-PNA 5612 even at 2× MIC concentration (4 µM), and only a minimal effect at 8 µM, in sharp contrast to the effect of colistin (Figure 7).

### 3.6. Effect on Target mRNA

**Figure 8:**
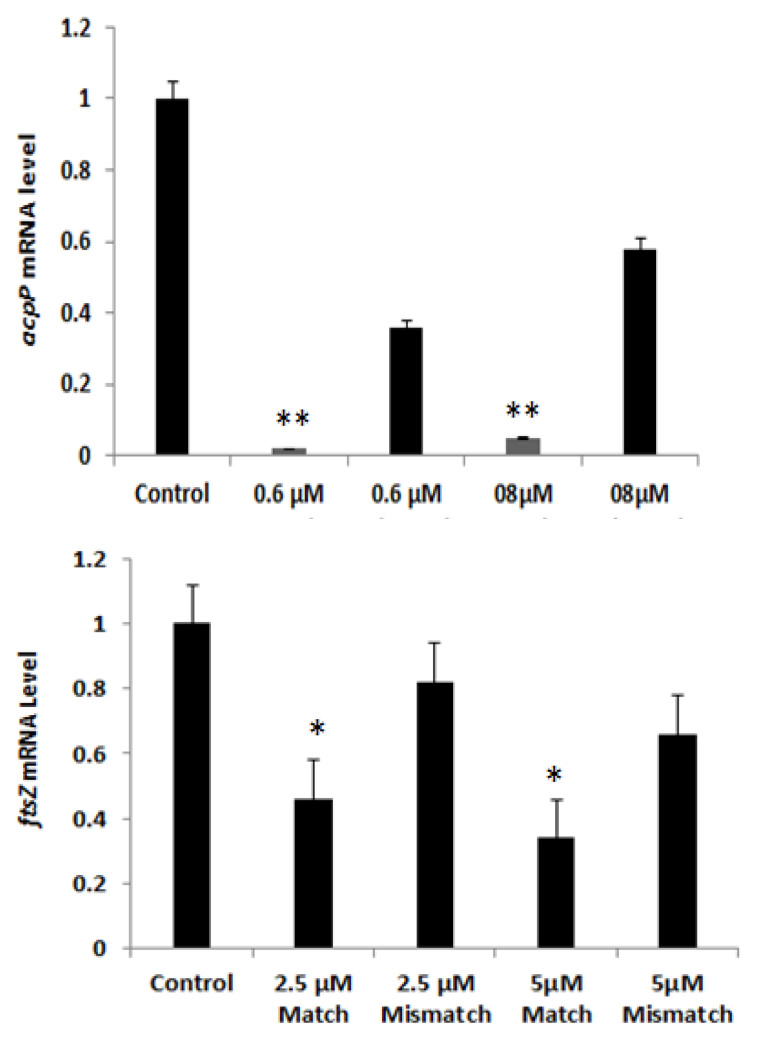
The *acpP* mRNA level in the CRAB AC44 strain after one hour treatment with anti-*acpP* (KFF)_3_K-PNA 5639 (match) or anti-*acpP* (KFF)_3_K-PNA 5640 (mismatch) compared with the untreated control. Gene expression levels were normalized to 16S rRNA through -∆Ct analysis and are presented as fold change versus control (untreated sample) calculated by the-∆∆Ct method. The *ftsZ* mRNA level in the CRAB AC44 strain after one hour treatment with anti-*ftsZ* (KFF)_3_K-PNA 5612 (match) or anti-*ftsZ* (KFF)_3_K-PNA 5615 (mismatch) compared with the untreated control. Error bars depict standard error of the mean. The presented data represents averages of triplicate determinations, performed at least two times. Comparison of each treatment with a control was done via two-tailed Student’s *t*-test (* = *p* ≤ 0.05, ** = *p* ≤ 0.01). As well, a comparison between BPP-PNA match and BPP-PNA mismatch with an untreated sample was performed via a one way ANOVA.

The molecular antisense mechanism of PNA oligomers in bacteria is believed to rely on translation inhibition via steric blockage of ribosome assembly on the mRNA. In other words, antisense PNA silencing in bacteria leads to translation repression and presumably unmasking of the mRNA. The mRNA unmasking may in turn lead to (nonsense mediated) decay [24]. In order to address whether such a mechanism is involved in the activities observed with the present BPP-PNAs, mRNA levels for the target genes were determined by RT-real time PCR. Specifically, the impact of different concentrations of anti-*acpP* (KFF)_3_-K-PNA 5639 and the corresponding mismatch control 5640 on the expression level of the *acpP* mRNA was analyzed (Figure 8). A significant reduction in the level of the *acpP* mRNA was observed upon treatment with BPP-PNA 5639 (>34-fold reduction) (Figure S6-A), while only a minor reduction was observed with the corresponding mismatch control BPP-PNA 5640 (4-fold reduction) (Figure S6-B). The differences in the relative expression of the *acpP* gene upon treatment with BPP-PNA 5640 was not statistically significant (*p*-value = 0.059, two-tailed Student’s *t*-test). We also find a reduction (up to 60%) in *ftsZ* mRNA 1 h after treatment with anti-*ftsZ* (KFF)_3_K-PNA 5612 (match) in the AC44 strain, while only a minor reduction was observed with the corresponding mismatch control PNA (up to 28%, Figure 8).

## 4. Discussion

The present study has identified three antisense (KFF)_3_-K-PNA conjugates targeting the translation initiation site of the mRNA of the *acpP* (PNA5639), *ftsZ* (PNA5612), and *rne* (PNA5656) genes, respectively, which exhibit bactericidal activity against carbapenem-resistant *A. baumannii* at 4 μM, while showing no significant cytotoxicity in HepG2 cells at 20 μM. An antisense mechanism of action was corroborated by the significantly lower activity of double mismatch controls, reduction of target mRNA levels upon PNA treatment, as well lack of membrane disruption activity. In addition, analogous BPP-PNAs based on the D-form (KFF)_3_-K peptide or the frequently used arginine rich (R-Ahx-R)_4_ showed inferior antibacterial activity. Finally, the anti-*ftsZ* PNA5612 was active against 12 CRAB clinical isolates.

Previously, several essential as well as non-essential genes have been targeted by BPP-PNA oligomers in *A. baumannii* [21,26,27,28]. Rose et al. [28] tested a (RXR)_4_XB-*carA* PNA oligomer (targeting *carA* as an essential gene) against four clinical strains of MDR-*A. baumannii* in minimal medium (M9). The MIC results demonstrated that all four strains were inhibited at a concentration of 1.25 µM (the low MIC most probably is related to the use of M9 minimal medium, as discussed below). In addition, in vivo testing of the BPP-PNA oligomer was done using a *Galleria mellonella* model of sepsis caused by one of the clinical strains. Unfortunately, this study suffers from the absence of an antisense mismatch control, and it is therefore not possible to ascribe the effects to an antisense mechanism of action. Guitian et al. [26] in another study found that the *lpxB* gene is a possible target for *A. baumannii*. They showed that a (KFF)_3_K-*lpxB* PNA inhibiting *lpxB* expression is bactericidal and found that this PNA showed higher antimicrobial activity in M9 compared to LB medium and ascribed the enhanced activity in M9 to the lower bacterial growth rate due to nutrient limitations in the M9 medium. We observed that using the M9 medium (with added glucose), overnight cultures only reached OD_595_ between 0.1 to 0.2, and therefore considered this unsuitable for MIC analyses.

Geller et al. [21] examined the effects of BPP-PMO oligomers targeting several essential genes (*acpP*, *ftsZ*, and *rpsJ*) against two ATCC strains (ATCC17978 and ATCC19606) and three clinical strains (AYE, M9, and AB0057) of *A. baumannii*. The most active anti *acpP* PMO was an R-Ahx conjugate (R-Ahx-R)_4_-Ahx-*acpP*; 08-0163 in the study) which exhibited MIC values of 8 µM (for ATCC strains) and 2 to 4 µM for clinical strains of *A. baumannii*. It is noteworthy that this PMO was designed to target the ATG codon at position +25 in the present database annotation (see Figure 1) [21], and therefore probably is not optimal for translation inhibition. Additionally, the mammalian cellular toxicity (in vitro) of the (R-Ahx-R)_4_-Ahx-*acpP*-PMO was not addressed, and a general, random sequence control instead of a specific mismatch control was used to demonstrate target specificity. In vivo experiments in a pulmonary infection model using intranasal administration showed efficacy of the compound, but significant effect was also observed for the random sequence control [21].

In our study, we selected new regions of *acpP* mRNA in accordance with the newly annotated translation initiation site(s) in the *A. baumannii acpP* gene and find that a KFF-based anti *acpP*-PNA which has not been studied before in *A. baumannii* shows higher antibacterial activity than the (R-Ahx-R)_4_-Ahx-*acpP*-PMO reported by Geller study [21]. For instance, the MIC of most active anti *acpP* compound in Geller study against ATCC19606 was 8 µM, while in our study, the MIC of the (KFF)_3_-K-eg1-*acpP* PNA (5639) against ATCC19606 was 2 µM.

In the case of the *ftsZ* gene, Geller et al. [21] studied only one region of the *ftsZ* mRNA (+4 to +14 (see Figure 3)) using an (RFF)_3_R-PMO. The MIC values of this PMO compound ranged from 16 to 64 µM against different strains of *A. baumannii* (because of the high MIC value, they did not report further studies on the compound). In the present study, antimicrobial activity optimization of several BPP-PNA oligomers targeting different regions of the *ftsZ* gene (near the start codon and RBS) resulted in an anti *ftsZ* PNA with MIC values ranging from 2 to 8 µM against a broad panel of clinical CRAB isolates. Surprisingly, the analogous (R-Ahx-R)_4_-Ahx PNA (5613) did not show significant antibacterial activity.

RNase E, coded for by the *rne* gene, is a central enzyme in RNA processing and metabolism [23,29], and therefore constitute a possible and interesting, but unexplored target for future precision antisense antibiotics. The present results demonstrate that antimicrobial anti-*rne* BPP-PNAs with MIC values of 2 μM against CRA can be found and may indeed be worth pursuing further.

Unfortunately, no systematic studies are yet available comparing directly the efficacy of fully analogous PNA and PMO antibacterial agents. Similarly, systematic comparison of the individual BPP carrier efficiency in different bacterial species is not available either. However, several studies have clearly shown that different bacterial species do respond differently to BPPs. For instance, the (KFF)_3_K peptide which is effective in e.g., *E. coli* as well as *A. baumannii* (as shown here) is not efficient in *P. aeruginosa* [17], whereas the (R-ahx-R)_4_ peptide is a potent BPP in both *E. coli* and *P. aeruginosa*. Likewise, analogous BPP/bacteria activity differences have been reported for *Burkholderia* versus *Pseudomonas* and *Acinetobacter* [30,31,32]. Furthermore, some peptides seem to work better in Gram-positive than in Gram-negative species [32], as can be expected from the significantly different envelope structures, Finally, although the PNA has extremely high biostability, the peptides are much less stable as exemplified by the (KFF)_3_K peptide, which has a half-life of less than 5 min in exponential bacterial culture [33]. This undoubtedly will vary with bacterial strain, medium, and bacterial density, and should also be taken into consideration when comparing and evaluating BPPs.

## 5. Conclusions

In conclusion, a series of new antisense PNA-peptide conjugates targeting translation of essential genes (*acpP*, *ftsZ*, and *rne*) have been identified and characterized in terms of low micromolar in vitro antibacterial activity against clinical isolates of carbapenem-resistant *Acinetobacter baumannii*. Therefore, these results provide new gene sequence targets and antisense BPP-PNA conjugate hit compounds for possible future development of precision antibiotics for treatment of infections by carbapenem-resistant *Acinetobacter baumannii*. The use of precision antibiotics will require genetically-based (PCR) diagnostics, and because predominantly the target pathogen is affected, any broad resistance development should be minimized. Further optimization of the compounds in the preclinical studies includes systematic gene walk around the identified target, also including target length variation [16]. In addition, structure activity relation (SAR) studies in terms of the delivery peptide part are required in order to optimize both antibacterial potency, but especially pharmacokinetics and pharmacodynamics (including in vivo toxicity) in appropriate animal (mouse) infection models.

## Figures and Tables

**Table 1:** PNAs targeted to the *acpP* gene sequence of *A. baumannii* AYE are displayed with their designation numbers. The MIC values (µM) for the four CRAB strains are presented compared to that for the standard, ATCC 19606 strain. m: Match, mm: Mismatch. * in Sigma MHB medium without cation. ** in Oxoid MHB medium, cation adjusted. ^a^ According to the start codon of the AYE, SDF strains. ^b^ According to the start codon of the ACICU, ATCC17978 strains. The gene sequence obtained from GenBank database updated January 2019. ^c^ D-form peptide. The PNAs are written from their N to C termini, and the N terminus corresponds to the 5′ end of a conventional oligonucleotide. K: lysine. F: phenylalanine. eg1: 8-amino-3,6-dioxaoctanoyl.

PNA No.	Sequence N→C	Target Position	Peptide				MIC (µM)	
				ATCC 19606	AC44	AC46	SSI 1104	AC2
5652 (m) *	ATATCGCTCAC	+40 to +50 ^b^	H-(KFF)_3_K-eg1	4	4	4	4	4
5653 (mm) *	ATACCGCTTAC		H-(KFF)_3_K-eg1	4	4	4	4	4
5639 (m) *	TGATTTGCCAC	+1 to +11 ^a^	H-(KFF)_3_K-eg1	2	1	1	2	1–2
5640 (mm) *	TGACTTGTCAC		H-(KFF)_3_K-eg1	4	4	4	4	4
5639 (m) **	TGATTTGCCAC	+1 to +11 ^a^	H-(KFF)_3_K-eg1	4	4	4	4	4
5640 (mm) **	TGACTTGTCAC		H-(KFF)_3_K-eg1	4	4	4	4	4
5823 (m) *	TGATTTGCCAC	+1 to +11 ^a^	H-(kff)_3_k-eg1 ^c^	4	4	4	4	4
5824 (mm) *	TGACTTGTCAC		H-(kff)_3_k-eg1 ^c^	8	4	4	8	8

**Table 2:** PNAs targeted to the *ftsZ* gene of *A. baumannii* AYE are displayed with their designation numbers. Minimum inhibitory concentrations (µM) of anti-*ftsZ* CPP-PNAs and mismatch control against CRAB strains. MHB without cation (Oxoid) was used. m: Match, mm: Mismatch. The PNAs are written from their N to C termini, and the N terminus corresponds to the 5′ end of a conventional oligonucleotide. K: lysine, F: phenylalanine. R: arginine. Ahx: 6-aminohexanoyl. eg1: 8-amino-3,6-dioxaoctanoyl. nd: not determined.

PNA No	Sequence N→C	Target Position	Peptide		MIC (µM)	
				ATCC 19606	AC44	SSI1104
5612 (m)	GAGGCCATGAC	−3 to +8	H-(KFF)_3_K-eg1	4	8	4
5615 (mm)	GAGTCCAGGAC		H-(KFF)_3_K-eg1	8	16	16
5613 (m)	GAGGCCATGAC	−3 to +8	H-(R-Ahx-R)_4_-Ahx	>16	>16	nd
5616 (mm)	GAGTCCAGGAC		H-(R-Ahx-R)_4_-Ahx	>16	>16	nd
5657 (m)	ATGAGGCCATG	−1 to +10	H-(KFF)_3_K-eg1	>16	16	nd
5658 (m)	GGCCATGACCT	−5 to +6	H-(KFF)_3_K-eg1	4	8	8

**Table 3:** Minimum inhibitory concentrations of the most potent Anti-*ftsZ* CPP-PNA (5612) and mismatch control (5615) against the CRAB and ATCC 19606 strains. MHB without cation (Oxoid) was used.

Strains	H-(KFF)_3_K-eg1-*ftsZ* PNA(5612)	H-(KFF)_3_K-eg1-*ftsZ* PNA(5615)
ATCC19606	4 µM	8 µM
AC1	4 µM	16 µM
AC2	2 µM	16 µM
AC3	4 µM	8 µM
AC4	8 µM	16 µM
AC5	4 µM	16 µM
AC6	4 µM	8 µM
AC7	4 µM	16 µM
AC8	8 µM	16 µM
AC9	2 µM	16 µM
AC44	8 µM	16 µM
AC46	8 µM	16 µM
SSI1104	4 µM	16 µM
MIC50	4 µM	16 µM

**Table 4:** PNAs targeted to the start-codon region of the *rne* gene of *A. baumannii* AYE are displayed with their designation numbers. The MIC values (µM) for the four CRAB strains are presented compared to that for the standard ATCC19606 strain. m: Match, mm: Mismatch. The PNAs are written from the N to C termini, and the N terminus corresponds to the 5′ end of a conventional oligonucleotide. K: lysine. F: phenylalanine. eg1: 8-amino-3, 6-dioxaoctanoyl. nd: not determined.

PNA No	Sequence N→C	Target Position	Peptide			MIC (µM)		
				ATCC 19606	AC44	AC46	SSI 1104	AC2
5637 (m)	ACGTTTCATGG	−2 to +9	H-(KFF)_3_K-eg1	1	1	1	nd	nd
5638 (mm)	ACGATTCTTGG		H-(KFF)_3_K-eg1	2	2	1	nd	nd
5654 (m)	ATACGTTTCAT	+1 to +11	H-(KFF)_3_K-eg1	2	1	2	2	2
5821 (mm)	ATCCGTTTAAT		H-(KFF)_3_K-eg1	4	1	2	2	2
5656 (m)	TTTCATGGGTG	−5 to +6	H-(KFF)_3_K-eg1	2	2	2	2	2
5822 (mm)	TTTGATGTGTG		H-(KFF)_3_K-eg1	8	8	8	8	8
5655 (m)	GTTTCATGGGT	−4 to +7	H-(KFF)_3_K-eg1	4	8	nd	nd	8

**Table 5:** Antibacterial activity of the most active compounds, anti-*ftsZ* (KFF)_3_K-PNA and anti-*acpP* (KFF)_3_K-PNA, against both target and non-target species. *^#^* In addition, the antibacterial activity of compound 3965 (designed against the *ftsZ* gene in *E. coli* with two base mismatches) against ATCC and CRAB strains is shown here. MHB without cation (Oxoid) was used. * Conjugated to H-(KFF)_3_K-eg1- at the N-terminal. m: Match. mm: Mismatch. nd: not determined.

Target	PNA	Sequence *	MIC (µM)	MIC (µM)	MIC (µM)	MIC (µM)
			*P. aeruginosa* (PAO1)	*E. coli K-12 MG1655*	*A. baumannii* ATCC *19606*	*A. baumannii* SSI1104
*ftsZ* (m)	5612	GAGGCCATGAC	>16 µM	>16 µM	4 µM	4 µM
*acpP* (m)	5639	TGATTTGCCAC	>16 µM	>16 µM	2 µM	2 µM
*acpP* (m)	5652	ATATCGCTCAC	16 µM	16 µM	4 µM	4 µM
*ftsZ* (mm) *^#^*	3965	TTCTAACAAAGT	nd	16 µM	16 µM	≥16 µM

## Data Availability

Not applicable.

## Supplementary Materials

The following are available online at https://www.mdpi.com/article/10.3390/biomedicines9040429/s1, Multiple sequence alignment of *ftsZ* and *rne* genes, Figure S2: A multiple sequence alignment of *acpP* gene, Figure S3: A multiple sequence alignment of AcpP proteins, Figure S4: The sequence of the *acpP* gene in *A. baumannii* and the other gram negative bacteria, Figure S5: Growth inhibition activity curve of the most active compounds against CRAB strains, Figure S6: Log2-fold change values determined by qPCR for *acpP* mRNA, Figure S7: Analytical HPLC and MALDI-TOF mass spectrometry results of the most active BPP-PNAs, Table S1: Clinical, phenotypic and genotypic characteristics of the CRAB in this study, Table S2: *Acinetobacter baumannii* strains that were examined for designing antisense in this study, Table S3: Anti-*ftsZ* BPP-PNA used in this study, Table S4: Anti-*acpP* BPP-PNA used in this study, Table S5: Anti-*rne* BPP-PNA used in this study, Table S6: Primers used in real time RT-PCR.
