# Supplementary material for: Targeting of the Essential acpP, ftsZ, and rne Genes in Carbapenem-Resistant Acinetobacter baumannii by Antisense PNA Precision Antibacterials
Source: Biomedicines. 2021 Apr 15;9(4):429. doi: 10.3390/biomedicines9040429 (PMC8071358; doi:10.3390/biomedicines9040429)
Supplement: Supplementary file 1 [file biomedicines-09-00429-s001.zip › biomedicines-1167280-supplementary.pdf]

biomedicines

MDPI

Article

Targeting of the Essential *acpP, ftsZ,* and *rne* Genes in Carbapenem-Resistant *Acinetobacter baumannii* by Antisense PNA Precision Antibacterials

Alireza Japoni Nejad [1,2], Nader Shahrokhi [2] and Peter E. Nielsen [1,*]

Supplementary

Supplementary Tables and Figures:

Table S1. (**A**) Clinical and phenotypic characteristics of the CRAB in this study (MIC µg/mL). (**B**) Genotypic characteristics of the CRAB strains in this study. The production of carbapenemases is an important mechanism responsible for the carbapenem resistance in *A. baumannii*. The genes encoding carbapenem-hydrolyzing class D ß-lactamases (CHDLs), class B metallo-ß-lactamases (MBLs) are shown here.

(A)

Clinical strains.	Source	Imipenem	Meropenem	Colistin	Ciprofloxacin	Tigecycline	Tetracycline	Ceftazidime	Gentamicin
**AC1**	Wound	8	4	1	8	0.5	16	64	8
**AC2**	Wound	16	16	1	16	1	64	64	16
**AC3**	Sputum	32	4	1	8	0.5	16	64	8
**AC4**	Trachea	8	16	1	16	1	64	64	16
**AC5**	Urine	16	16	1	16	1	64	32	16
**AC6**	Blood	16	16	1	32	1	16	32	16
**AC7**	Blood	16	16	1	16	1	64	64	16
**AC8**	Sputum	16	16	1	32	1	32	64	16
**AC9**	CSF	16	16	1	8	1	32	32	16
**AC44**	Urine	16	16	0.5	32	1	256	256	16
**AC46**	Sputum	16	16	1	32	0.5	128	128	16
**SSI1104**	NA	NA	> 16	1	> 8	1	64	> 8	32
**ATCC 19606**	Type strain	1	1	1	1	0.5	1	8	4

(B)

Clinical Isolates No	*bla*$_{IMP}$	*bla*$_{VIM}$	*bla*$_{SPM}$	*bla*$_{GES}$	*bla*$_{OXA23}$	*bla*$_{OXA-24}$	Is/ *bla*$_{OXA23}$	Is/*bla*$_{oxa51}$
**AC1**	_	+	+	+	+	_	_	_
**AC2**	_	+	_	+	+	_	+	+
**AC3**	+	+	_	_	+	+	_	_
**AC4**	_	+	_	_	+	+	+	_
**AC5**	_	+	_	_	+	+	+	+
**AC6**	_	+	_	_	+	+	+	+
**AC7**	_	+	_	+	+	+	+	+
**AC8**	_	+	_	+	+	+	+	+
**AC9**	_	+	_	+	+	+	+	+
**AC44**	_	+	_	+	+	+	+	+

**AC46**	_	+	_	_	+	+	+	+
**SSI1104**	ND	ND	ND	ND	+	ND	ND	ND

Table S2. *Acinetobacter baumannii* strains that were examined for designing antisense in this study. The target genes ID obtained from GenBank database updated January 2019.

**Strains**	***ftsZ* Gene Bank AC number**	***acpP* Gene Bank AC number.**
A. baumannii AYE	NC_010400.1	NC_010410.1
A.baumannii ATCC17978	A1S_3331	CP000521
A. baumannii ACICU	NC_010611.1	NC_010611.1
A. baumannii SDF	ABSDF3467	NC_010400.1
A.baumannii ATCC 19606	CP046654.1	CP046654.1

Table S3. Anti-*ftsZ* BPP-PNA used in this study.

		**Mass(found/calculate)**
**5612**	H-(KFF)$_3$K-eg1-GAGGCCATGAC-NH$_2$	4572(4568)
**5615**	H-(KFF)$_3$K-eg1-GAGTCCAGGAC-NH$_2$	4568(4568)
**5613**	H-(R-Ahx-R)$_4$-Ahx-GAGGCCATGAC-NH$_2$	4914(4917)
**5616**	H-(R-Ahx-R)$_4$-Ahx-GAGTCCAGGAC-NH$_2$	4914(4917)
**5657**	H-(KFF)$_3$K-eg1-ATGAGGCCATG-NH$_2$	4583(4580)
**5658**	H-(KFF)$_3$K-eg1-GGCCATGACCT-NH$_2$	4583(4580)

Table S4. Anti-*acpP* BPP-PNA used in this study.

		**Mass(found/calculated)**
**5652**	H-(KFF)$_3$K-eg1-ATATCGCTCAC-NH$_2$	4478(4477)
**5653**	H-(KFF)$_3$K-eg1-ATACCGCTTAC-NH$_2$	4478(4477)
**5639**	H-(KFF)$_3$K-eg1-TGATTTGCCAC-NH$_2$	4512(4509)
**5640**	H-(KFF)$_3$K-eg1-TGACTTGTCAC-NH$_2$	4510(4509)
**5823**	H-(kff)$_3$k-eg1-TGATTTGCCAC-NH$_2$	4509(4507)
**5824**	H-(kff)$_3$k -eg1-TGACTTGTCAC-NH$_2$	4509(4507)

Table S5. Anti-*rne* BPP-PNA used in this study.

		**Mass(found/calculated)**
**5637**	H-(KFF)$_3$K-eg1-ACGTTTCATGG-NH$_2$	4549(4540)
**5638**	H-(KFF)$_3$K-eg1-ACGATTCTTGG-NH$_2$	4549(4548)
**5654**	H-(KFF)$_3$K-eg1-ATACGTTTCAT-NH$_2$	4508(4507)
**5821**	H-(KFF)$_3$K-eg1-ATCCGTTTAAT-NH$_2$	4508(4509)
**5656**	H-(KFF)$_3$K-eg1-TTTCATGGGTG-NH$_2$	4580(4582)
**5822**	H-(KFF)$_3$K-eg1- TTTGATGTGTG -NH$_2$	4580(4580)
**5655**	H-(KFF)$_3$K-eg1-GTTTCATGGGT-NH$_2$	4580(4578)

Table 6. Primers used in Real time RT-PCR.

Primer	Genes	PCR Product
acpP F: AACAACTTGGTCTTAAAG<br>acpP R: GATTGAACTGTAGTGATT	acpP	163 bp
ftsZ F: CAGCACATGGTACAGTCT<br>ftsZ R: GGCATTCATACAGTCAAGTG	ftsZ	84 bp
zipA-F : GAGCAGATTATCGCATTA<br>zipA-R : ATACGCAATACAGAGAAC	zipA	170bp
polA-F : AACATTCCAATTCGTAGAC<br>polA-R : TGAACTAAGGCATCATCT	polA	143bp
16s rRNA-F: CAAACAGGATTAGATACC<br>16s rRNA-R: GTCAATTCATTTGAGTTT	16srRNA	146 bp

A

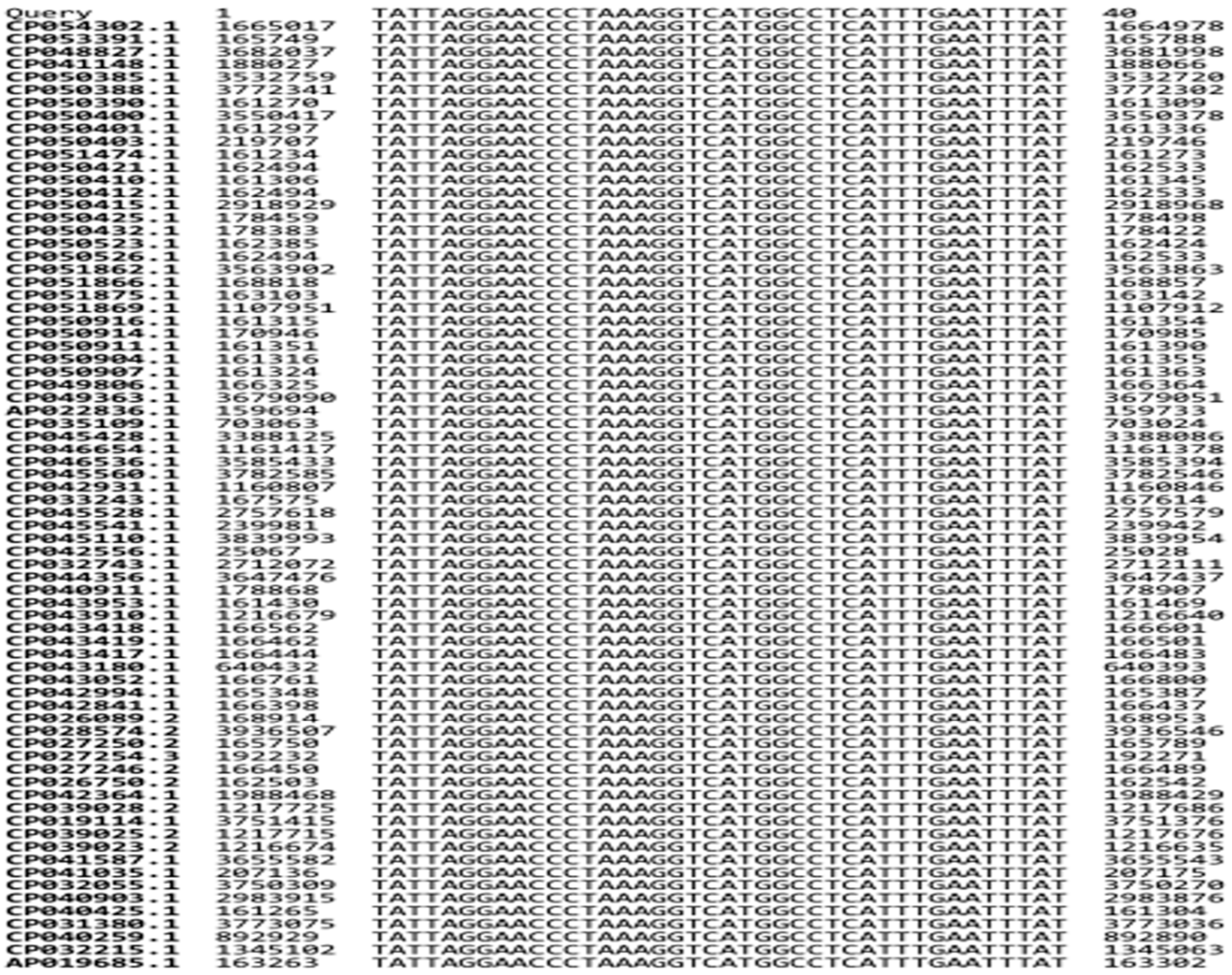

B

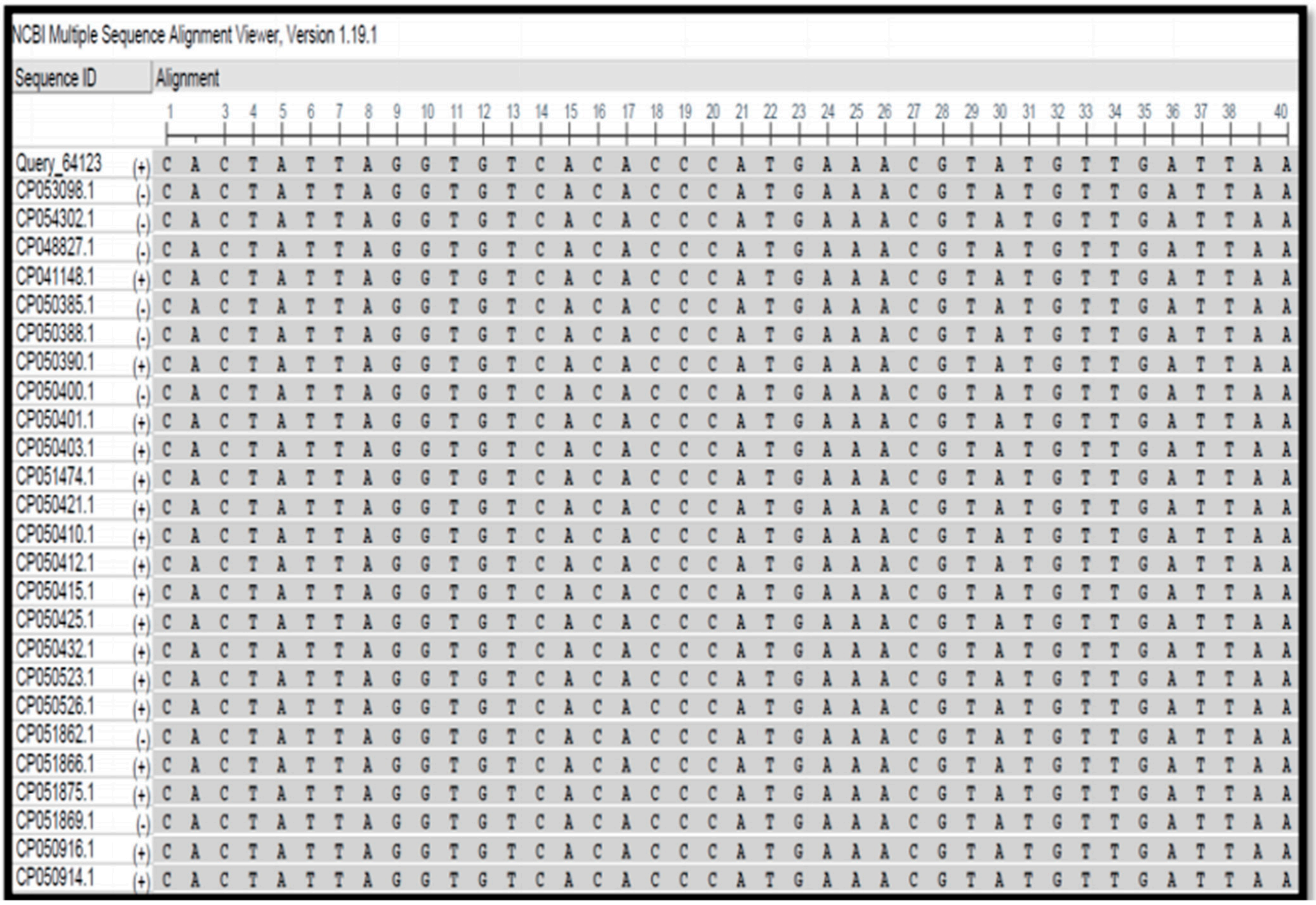

Figure S1. (**A**) Multiple sequence alignment of *ftsZ* gene, region -20 to +20, using Basic Local Alignment Search Tool (BLAST) on the NCBI website. The BLAST results confirmed the 5′ terminal region of the *ftsZ* gene including the ribosomal binding site and the start ATG codon is conserved among *A.baumannii* species. (**B**) Multiple sequence alignment of the rne gene, region -20 to +20, using BLAST on the NCBI website. The BLAST results confirmed the 5′ terminal region of the rne gene including the ribosomal binding site and the start ATG codon is conserved among *A.baumannii* species.

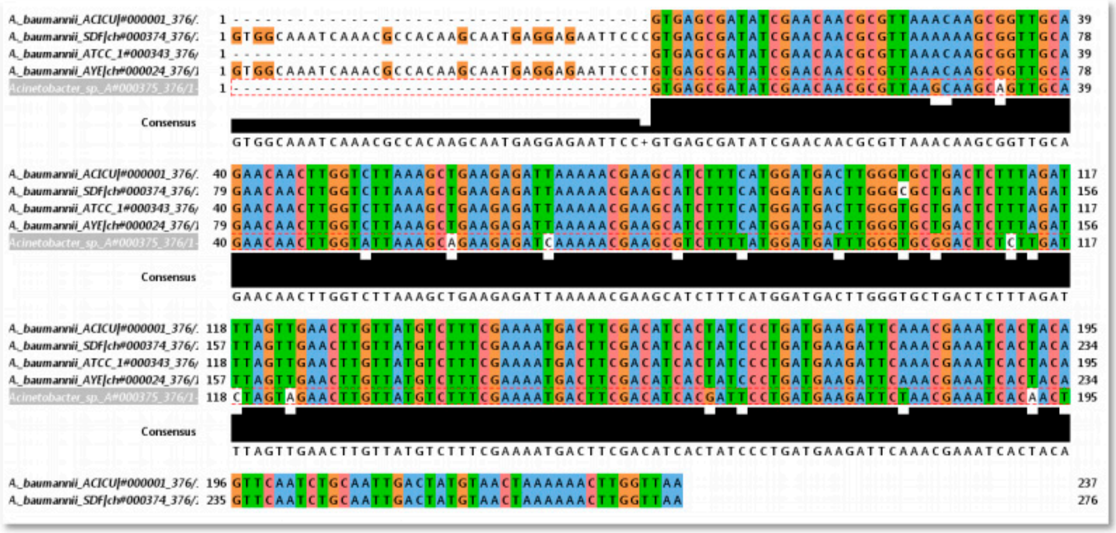

Figure S2. A multiple sequence alignment of *acpP* gene (GenBank database updated January 2019) using the Basic Local Alignment Search Tool (BLAST) on the NCBI website in *A. baumannii* AYE,*A.baumannii* SDF, *A. baumannii* ACICU, *A. baumannii* ATCC 17978 and *Acinetobacter* sp ADP1 (Sequence ID was represented in Table S2)[32]. The *acpP* gene has two lengths in different strains of *A.baumannii*.The *acpP* gene in *A.baumannii* ATCC19606, AYE and SDF strains was 276 bp in length while that was 237 bp in length on both ACICU and ATCC17978 strains.

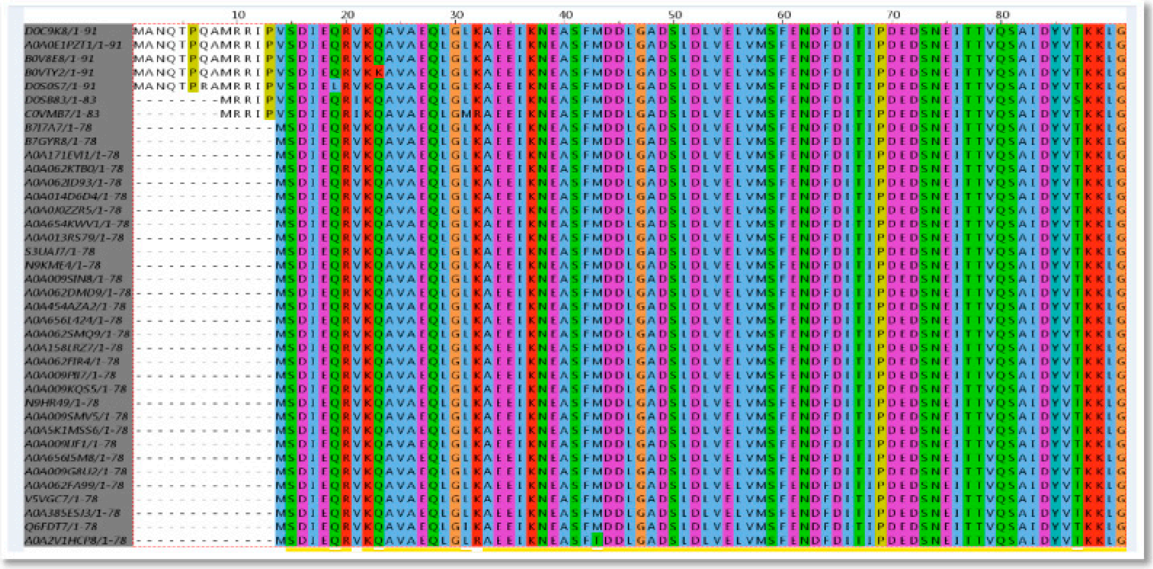

Figure S3. A multiple sequence alignment of AcpP proteins (UniProt updated 2019/01).using UniProt website (*A. baumannii* AYE UniProt ID: B0V8E8, *A. baumannii* SDF UniProt ID: B0VTY2 and *A. baumannii* ATCC 19606 UniProt ID: D0C9K8.

A

21 Genes

	Accession Number	Gene Name	Organism	Replicon	Gene Length	Location (kb)	Description
	ABAYE2991	*acpP*	*A. baumannii* AYE	*chromosome*	276	3018.68	Acyl carrier protein
	JW1080	*acpP*	*E. coli* W3110	*chromosome*	237	1153.19	Acyl carrier protein
	UTI89_C1220	*acpP*	*E. coli* UTI89	*chromosome*	237	1196.58	Acyl carrier protein
	SbBS512_E2230	*acpP*	*S. boydii* CDC 3083-94	*chromosome*	237	2030.10	Acyl carrier protein
	SBO_1969	*acpP*	*S. boydii* Sb227	*chromosome*	237	1943.72	Acyl carrier protein
	SCH_1144	*acpP*	*S. choleraesuis* enterica sv Choleraesuis SC-B67	*chromosome*	237	1260.17	Acyl carrier protein
	SeD_A2173	*acpP*	*S. dublin* sv Dublin CT_02021853	*chromosome*	237	2086.89	Acyl carrier protein
	SDY_2056	*acpP*	*S. dysenteriae* Sd197	*chromosome*	237	1868.37	Acyl carrier protein
	SEN1853	*acpP*	*S. enteritidis* P125109	*chromosome*	237	1958.59	Acyl carrier protein
	SFV_1114	*acpP*	*S. flexneri* 5 8401	*chromosome*	237	1157.41	Acyl carrier protein
	S1178	*acpP*	*S. flexneri* 2a 2457T	*chromosome*	237	1142.22	Acyl carrier protein
	CP0124	*acpP*	*S. flexneri* 2a 301	*pCP301*	237	107.17	Acyl carrier protein
	SF1098	*acpP*	*S. flexneri* 2a 301	*chromosome*	237	1139.33	Acyl carrier protein
	SG1926	*acpP*	*S. gallinarum* 287/91	*chromosome*	237	1991.33	Acyl carrier protein
	SPAB_02327	*acpP*	*S. paratyphi* enterica sv Paratyphi B SPB7	*chromosome*	237	1928.49	Acyl carrier protein
	SPA1655	*acpP*	*S. paratyphi* enterica sv Paratyphi A ATCC 9150	*chromosome*	237	1720.08	Acyl carrier protein
	SeSA_A1266	*acpP*	*S. schwarzengrund* sv Schwarzengrund CVM19633	*chromosome*	237	1238.41	Acyl carrier protein
	SSON_1114	*acpP*	*S. sonnei* Ss046	*chromosome*	237	1192.91	Acyl carrier protein
	STY1235	*acpP*	*S. typhi* CT18	*chromosome*	237	1189.78	Acyl carrier protein
	t1724	*acpP*	*S. typhi* enterica sv Typhi Ty2	*chromosome*	237	1791.40	Acyl carrier protein
	STM1196	*acpP*	*S. typhimurium* LT2	*chromosome*	237	1280.11	Acyl carrier protein

B

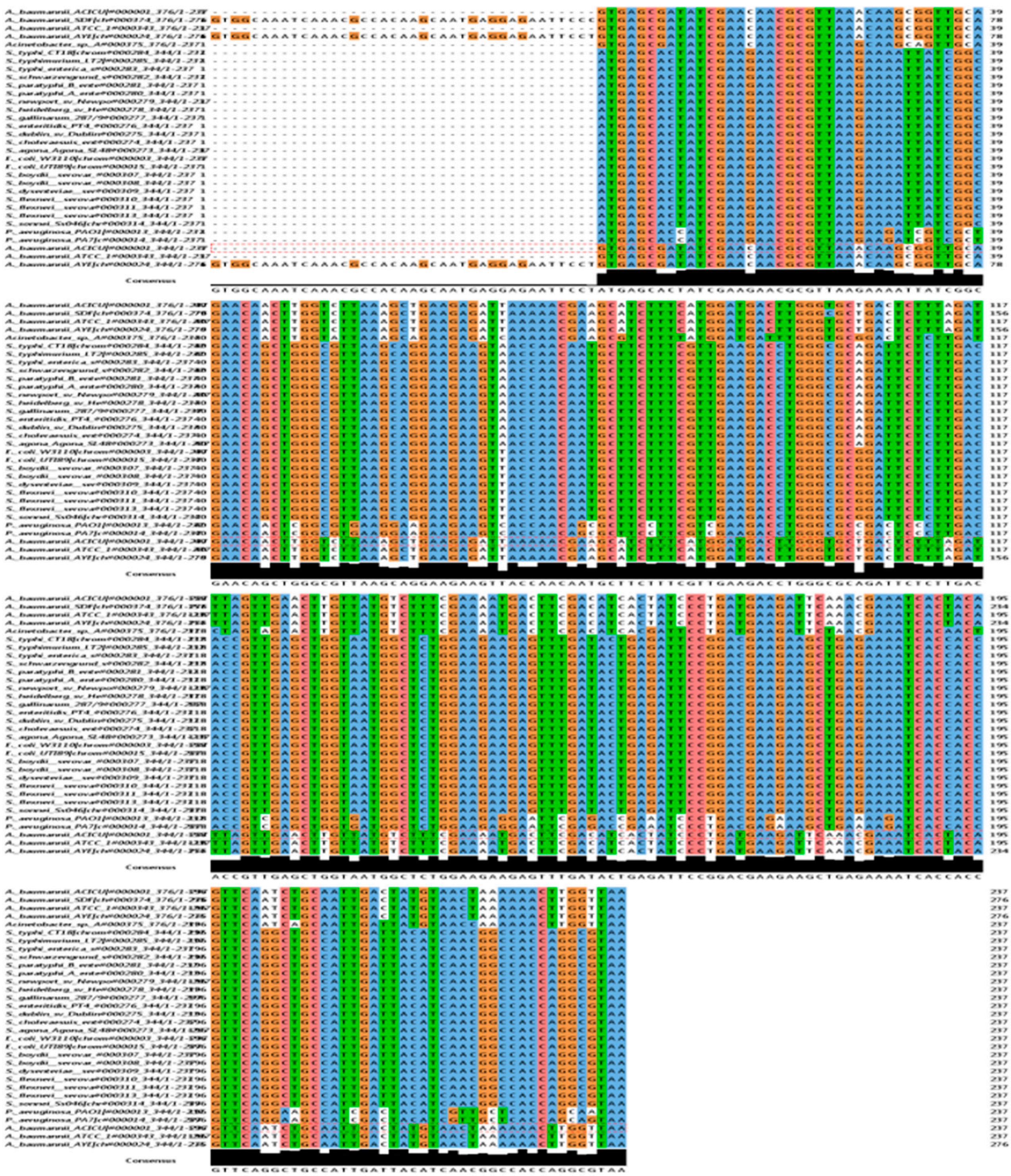

Figure S4. (**A**)The sequence of the *acpP* gene in *A.baumannii* and the other gram negative bacteria was obtained from genolist database[32] .(**B**)Multiple sequence alignment of *acpP* gene was done for determination of conserved regions.

(A)

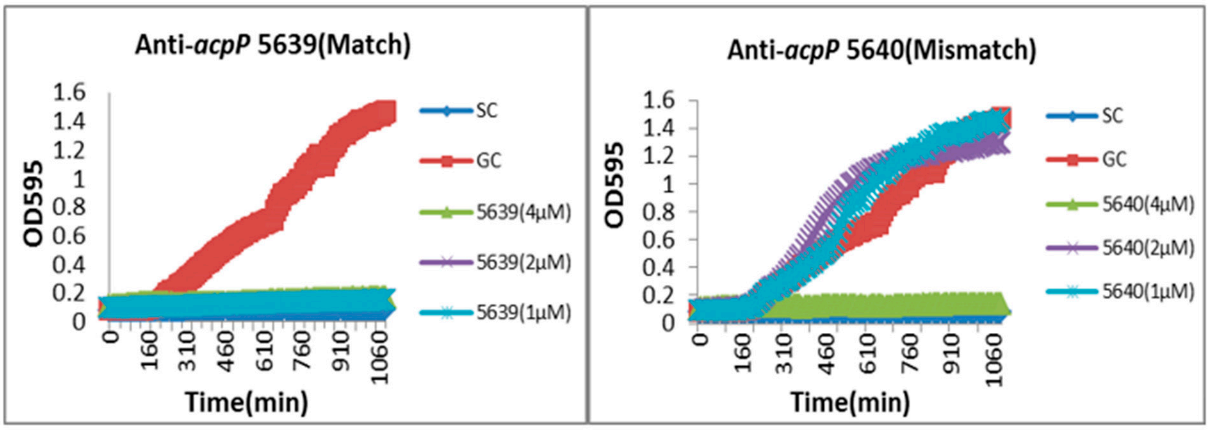
Anti-*acpP* 5639(Match)
OD595
Time(min)
SC
GC
5639(4μM)
5639(2μM)
5639(1μM)
Anti-*acpP* 5640(Mismatch)
5640(4μM)
5640(2μM)
5640(1μM)

(B)

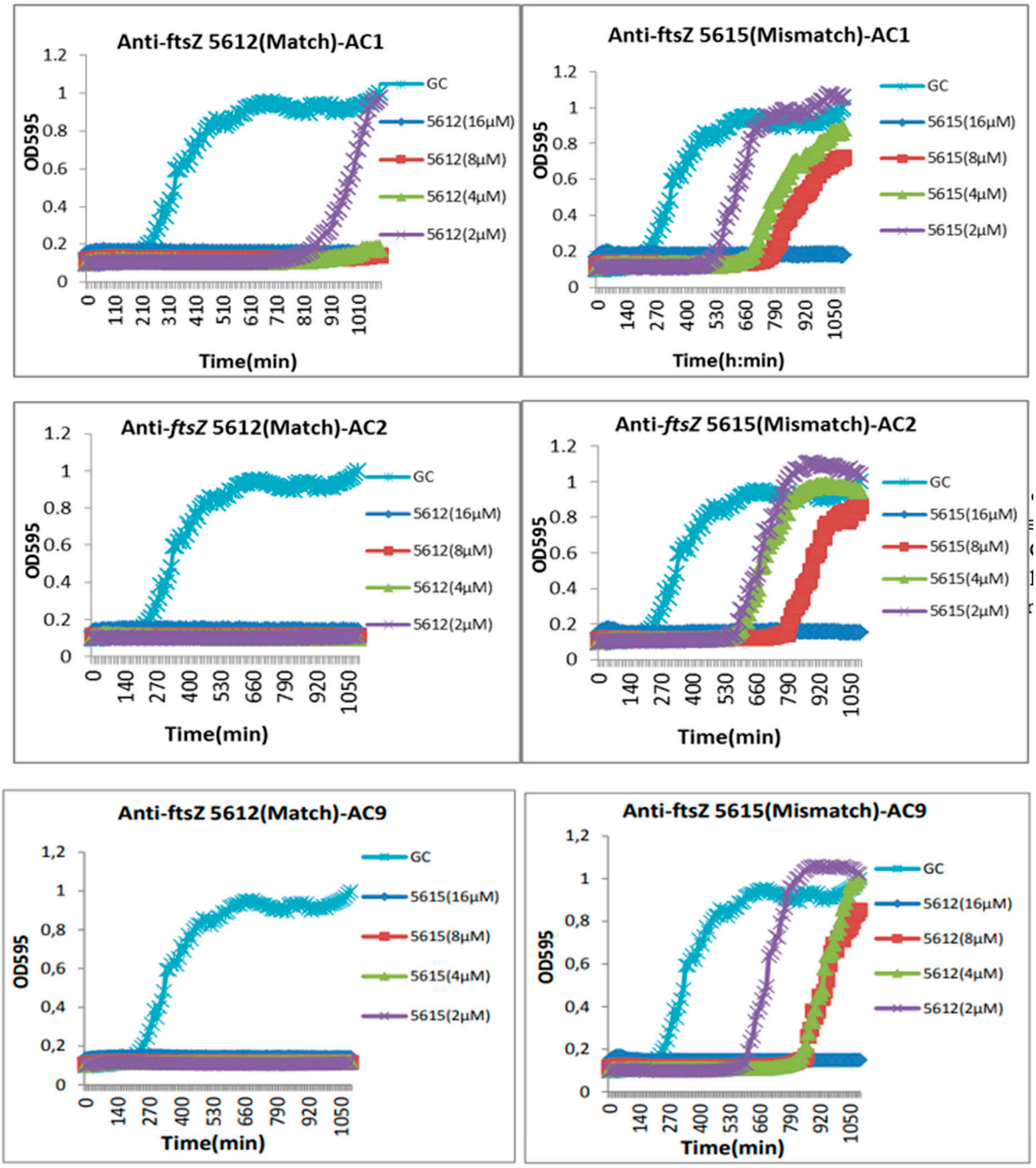

Figure S5. (**A**) Growth inhibition activity curve of the most active anti-*acpP* (KFF)$_3$K-PNA(5639) and a corresponding mismatch control (5640) against CRAB strain (AC44).(**B**) Growth inhibition activity of the most active anti-*ftsZ* (KFF)$_3$K-PNA(5612) and a corresponding mismatch control (5615) against three CRAB strains(AC1,AC2 and AC9). Concentration-dependent growth inhibition of anti-*ftsZ* (KFF)$_3$K-PNA(5612) and a corresponding mismatch controls on the growth of three CRAB strains (AC1,AC2 and AC9). Bacteria were grown in the absence of PNA (GC) and different doses of peptide–PNA conjugates (2µM to 16 µM).

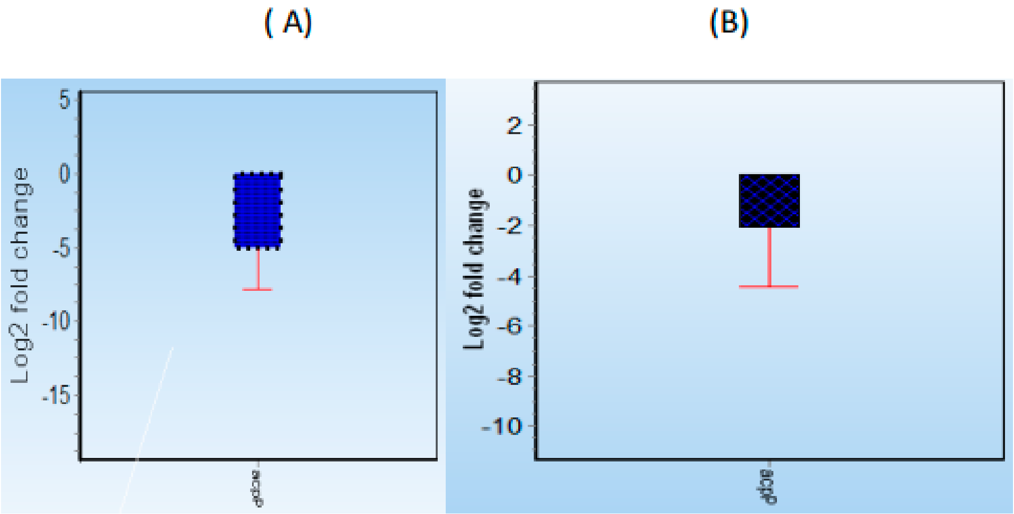

Figure S6. (**A**) Log2-fold change values determined by qPCR for *acpP* mRNA in CRAB AC44 strain, 1 hour treated with different concentration of anti-*acpP* (KFF)$_3$K-PNA 5639 (match) compared to the untreated sample. (B) Log2-fold change values determined by qPCR for *acpP* mRNA in CRAB AC44 strain, 1 hour treated with different concentration of anti-*acpP* (KFF)$_3$K-PNA 5640 (Mismatch) compared with the untreated sample. P values were calculated between (A) match and untreated sample (P-Value = 0.01) and (**B**) Mismatch and untreated sample (P-Value = 0.059) using twotailed Student's t-test (P<0.05 was considered as significant). Values shown in the graphs are the mean of three replicates. Error bars depict standard error of the mean.

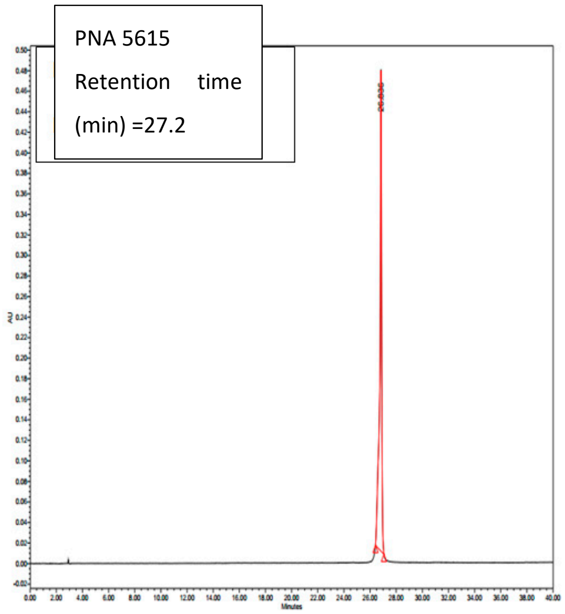
PNA 5615
Retention time
(min) =27.2
AU
Minutes

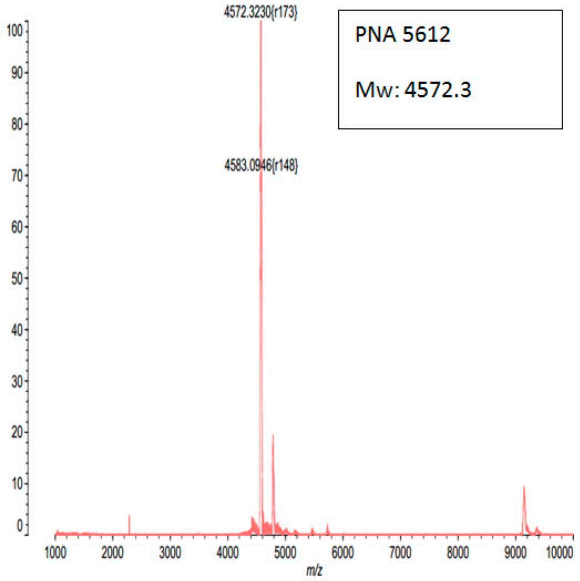
4572.3230(r173)
4583.0946(r148)
PNA 5612
Mw: 4572.3
m/z

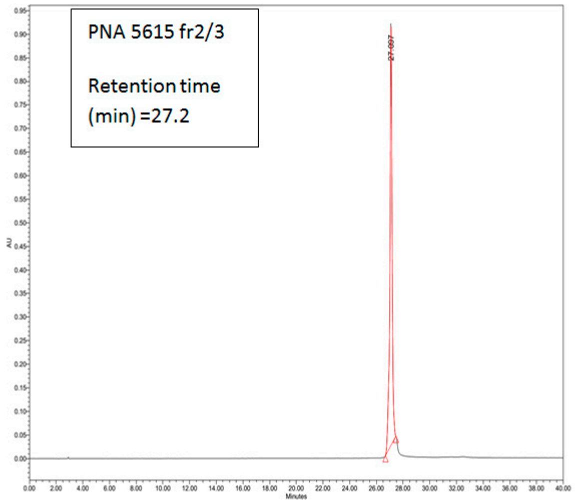
PNA 5615 fr2/3
Retention time
(min) =27.2
AU
Minutes

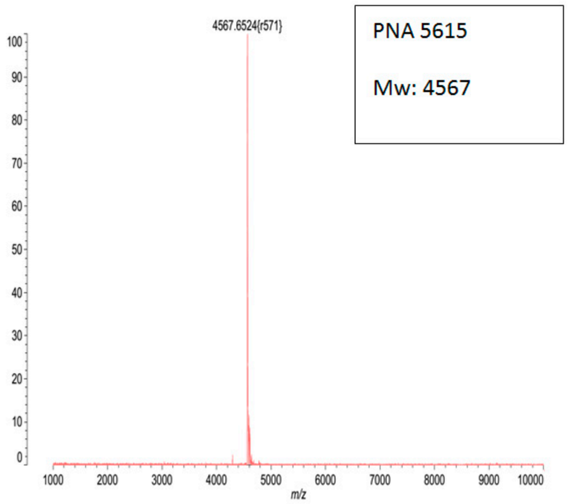
4567.6524(r571)
PNA 5615
Mw: 4567
m/z

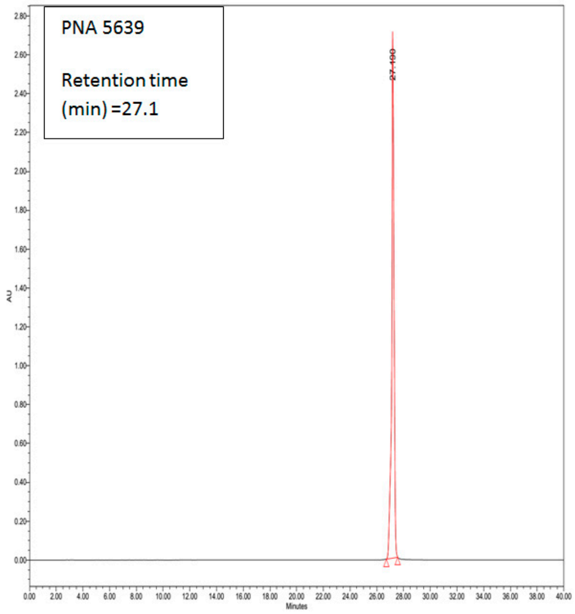
PNA 5639
Retention time (min) =27.1
27.190
AU
Minutes

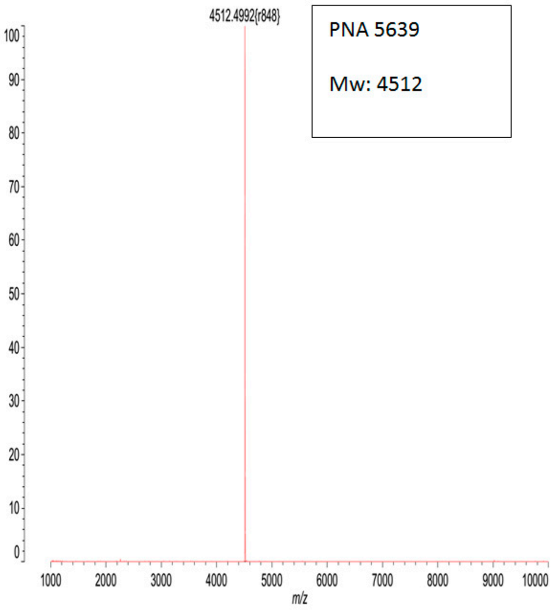
4512.4992(r848)
PNA 5639
Mw: 4512
m/z

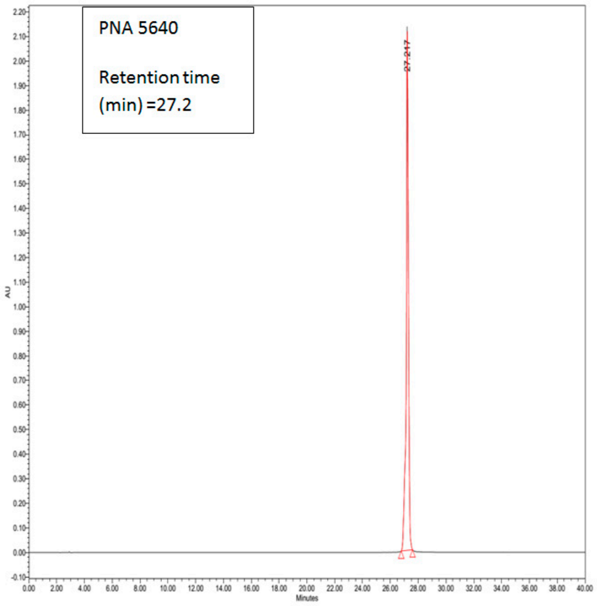
PNA 5640
Retention time (min) =27.2
27.217
AU
Minutes

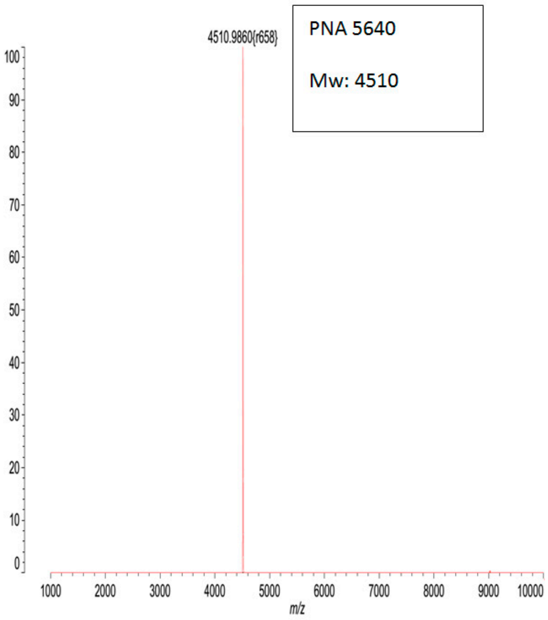
4510.9860(r658)
PNA 5640
Mw: 4510
m/z

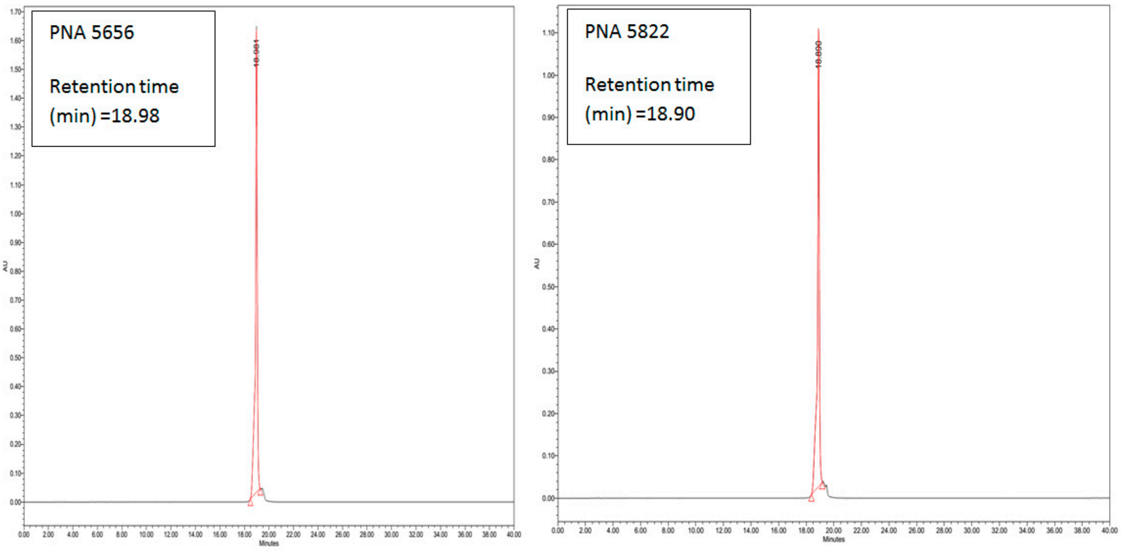

Figure S7. Analytical HPLC and MALDI-TOF mass spectrometry results of the most active BPP-PNAs.
